# Isogenic Pairs of Wild Type and Mutant Induced Pluripotent Stem Cell (iPSC) Lines from Rett Syndrome Patients as *In Vitro* Disease Model

**DOI:** 10.1371/journal.pone.0025255

**Published:** 2011-09-26

**Authors:** Gene Ananiev, Emily Cunningham Williams, Hongda Li, Qiang Chang

**Affiliations:** 1 Waisman Center, University of Wisconsin-Madison, Madison, Wisconsin, United States of America; 2 Stem Cell and Regenerative Medicine Center, University of Wisconsin-Madison, Madison, Wisconsin, United States of America; 3 Genetics Training Program, University of Wisconsin-Madison, Madison, Wisconsin, United States of America; 4 Department of Genetics and Neurology, University of Wisconsin-Madison, Madison, Wisconsin, United States of America; Wellcome Trust Centre for Stem Cell Research, United Kingdom

## Abstract

Rett syndrome (RTT) is an autism spectrum developmental disorder caused by mutations in the X-linked methyl-CpG binding protein 2 (*MECP2*) gene. Excellent RTT mouse models have been created to study the disease mechanisms, leading to many important findings with potential therapeutic implications. These include the identification of many MeCP2 target genes, better understanding of the neurobiological consequences of the loss- or mis-function of MeCP2, and drug testing in RTT mice and clinical trials in human RTT patients. However, because of potential differences in the underlying biology between humans and common research animals, there is a need to establish cell culture-based human models for studying disease mechanisms to validate and expand the knowledge acquired in animal models. Taking advantage of the nonrandom pattern of X chromosome inactivation in female induced pluripotent stem cells (iPSC), we have generated isogenic pairs of wild type and mutant iPSC lines from several female RTT patients with common and rare RTT mutations. R294X (arginine 294 to stop codon) is a common mutation carried by 5–6% of RTT patients. iPSCs carrying the R294X mutation has not been studied. We differentiated three R294X iPSC lines and their isogenic wild type control iPSC into neurons with high efficiency and consistency, and observed characteristic RTT pathology in R294X neurons. These isogenic iPSC lines provide unique resources to the RTT research community for studying disease pathology, screening for novel drugs, and testing toxicology.

## Introduction

Rett syndrome (RTT) is a debilitating autism spectrum developmental disorder that predominantly affects females [1], [2]. RTT patients often experience normal development for the first 6–18 months of their lives, which is followed by a rapid developmental regression between the ages of 1 to 3 years. Major symptoms of RTT include reduced head growth, social withdrawal, loss of previously acquired skills including purposeful hand use and expressive language, gait ataxia, stereotypic movement of the hands, and autonomic dysfunctions such as respiratory distress [3]. At an estimated prevalence of 1 in 10,000–15,000 girls, RTT is the second most common cause of X-linked mental retardation (XLMR).

Mutations in the X-linked methyl-CpG binding protein 2 (*MECP2*) gene were identified to account for almost all of the classic RTT cases [4], [5], [6], [7]. MeCP*2* is involved in modulating chromatin structure and gene transcription through its binding to methylated DNA [8]. To study RTT disease mechanisms, mouse models have been generated by engineering *Mecp2* gene deletions [9], [10], [11]. Male mutant mice that lack either the entire *Mecp2* gene [10] or the essential methyl-DNA binding domain [9] develop normally until 5 weeks of age when RTT-like symptoms are first observed, including reduced brain weight, hindlimb clasping and impaired locomotor function. These mice later develop respiratory abnormalities [12] and die prematurely around 8–10 weeks of age. Female mutant mice heterozygous for these deletions also display RTT-like symptoms, yet the onset is typically much later than in their male counterparts. At the cellular level, the most noticeable pathologies in these mice are the decrease in the size of neuronal nuclei [9] and the reduced complexity of neuronal dendritic arborization [13], [14], [15], [16], which are also observed in human autopsy samples [17].

Although they have been extremely useful in studying the molecular mechanism of RTT, mouse models may have limitations in mimicking human RTT mutations and in drug screening. To date, almost all the available RTT mouse models are loss-of-function alleles that either lacks the whole gene or harbors large deletions in the *Mecp2* gene [9], [10], [11]. In contrast, many human RTT patients carry missense mutations in the *MECP2* gene. Moreover, 92% of new drugs that pass preclinical tests, including tests on animals, fail to reach the market either because of safety or efficacy failures (*US FDA: Report on Challenge and Opportunity on the Critical Path to New Medical Products, March 2004*). This highlights the potential differences in the underlying biology between humans and common research animals. Therefore there is a need to establish cell culture-based human models for studying disease mechanisms to validate and expand the knowledge gained from animal models. Groundbreaking work from the Yamanaka group showed that it is feasible to generate induced pluripotent stem cells (iPSC) from mouse embryonic fibroblasts [18] and human fibroblasts [19] by retrovirus mediated expression of four key stem cell transcription factors: *OCT4*, *SOX2*, *c-Myc*, and *KLF4*. The Thomson group independently demonstrated that iPSCs could be generated from human adult fibroblasts [20] by lentivirus mediated expression of overlapping but different combination of stem cell factors: *OCT4*, *SOX2*, *NANOG*, and *LIN28*. These early studies have established the iPSC technology as a novel tool to model human diseases and screen for drugs. Since then, patient-specific iPSCs have been successfully generated for several diseases [21], [22], [23], [24], including Rett syndrome [25], [26], [27], [28], [29]. Taking advantage of the nonrandom pattern of X chromosome inactivation in female iPSCs [30], we have generated several novel isogenic pairs of wild type and mutant iPSC lines from patient fibroblasts carrying common RTT mutations, as well as from an unrelated healthy individual. We show here that all female RTT iPSC lines have highly skewed XCI pattern, and clonally express either the wild type or the mutant, but not both, alleles of the *MECP2* gene. Both the wild type and mutant RTT iPSC lines can be consistently and efficiently differentiated into neurons. For phenotypic characterization, we focused our study on the R294X neurons and their isogenic wild type controls, because iPSCs with this common RTT mutation have not been described previously. Our extensive analysis revealed that the R294X neurons were smaller than their isogenic controls, which is a characteristic RTT pathology observed in both RTT patients and *Mecp2* mutant mice.

## Results

### Generation and characterization of iPSC lines from RTT patient fibroblasts

**Figure 1:**
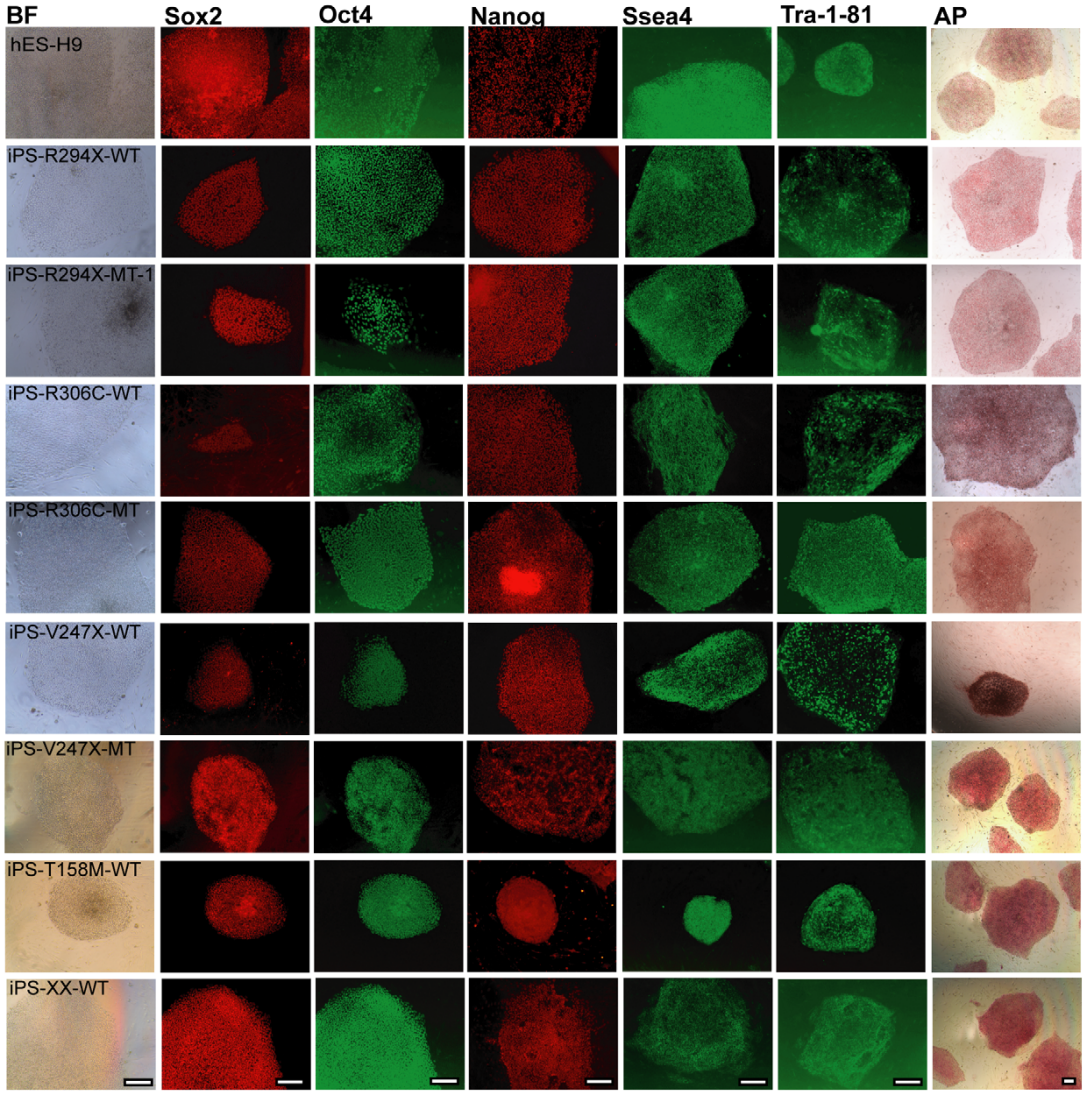
RTT iPSCs express the same set of pluripotency markers as previously characterized hESC and hiPSCs lines. Representative images of hESC (H9) and hiPSC colonies stained with antibodies against pluripotency markers Sox2, Oct4, Nanog, Ssea4, and Tra-1-81, and processed for alkaline phosphatase reaction. Each row represents colonies from one hESC or hiPSC line. iPS-XX-WT was derived from a healthy female. Each column represents one pluripotency marker. BF stands for bright field. Scale bars = 200 µm.

Three female RTT patient fibroblast lines, as well as 1 female fibroblast line from an unrelated healthy individual, were obtained from the Coriell Institute for Medical Research. Among these Coriell fibroblast lines, GM17880 was derived from 5-year-old female RTT patient carrying the T158M mutation (Threonine 158 to Methionine, missense, present in 9–11% of RTT patients), GM07982 was derived from a 25-year-old female patient carrying the V247X mutation (Valine 247 to stop codon, nonsense, very rare in RTT patients), GM11270 was derived from a 8-year-old female patient carrying the R306C mutation (Arginine 306 to Cysteine, missense, present in 4–7% of RTT patients), and AG07306 was derived from a 28-year old healthy female. In addition, fibroblast line RS0502 was derived at the Waisman Center of the University of Wisconsin-Madison from an 11-year-old female patient carrying the R294X (Arginine 294 to stop codon, nonsense, present in 5–6% of RTT patients). The exact location of each of these mutations in relation to known functional domains of MeCP2 protein is illustrated in Figure S1. iPSCs from all above described fibroblast lines were generated by simultaneous infection with either lentiviruses encoding *OCT4*, *NANOG*, *SOX2* and *LIN28* as described in [20] or retroviruses encoding *OCT4*, *SOX2*, *c-Myc* and *KLF4* [19]. 15–20 independent iPSC lines were established and expanded from each of the original fibroblast lines. Detailed characterization revealed that these iPSC lines had the characteristic human embryonic stem cell (hESC) morphology (Figure 1, leftmost column), carried the parental RTT mutations at the DNA level (Figure S2), expressed the same set of pluripotency markers as the hESCs did (Figure 1), had normal karyotypes (Figure S3), showed extensive demethylation in the promoter of the endogenous Oct4 locus (Figure S4), and formed teratomas when injected into NOD-SCID mice (Figure S5). In addition, retroviral expression of reprogramming factors was silenced in all R294X iPSC lines examined (Figure S6). For comparison purposes, a previously reported iPSC line, iPS-IMR90-4, and a hESC line, H9, were included in some of these experiments.

### X chromosome inactivation is not random in female RTT iPSC lines

**Figure 2:**
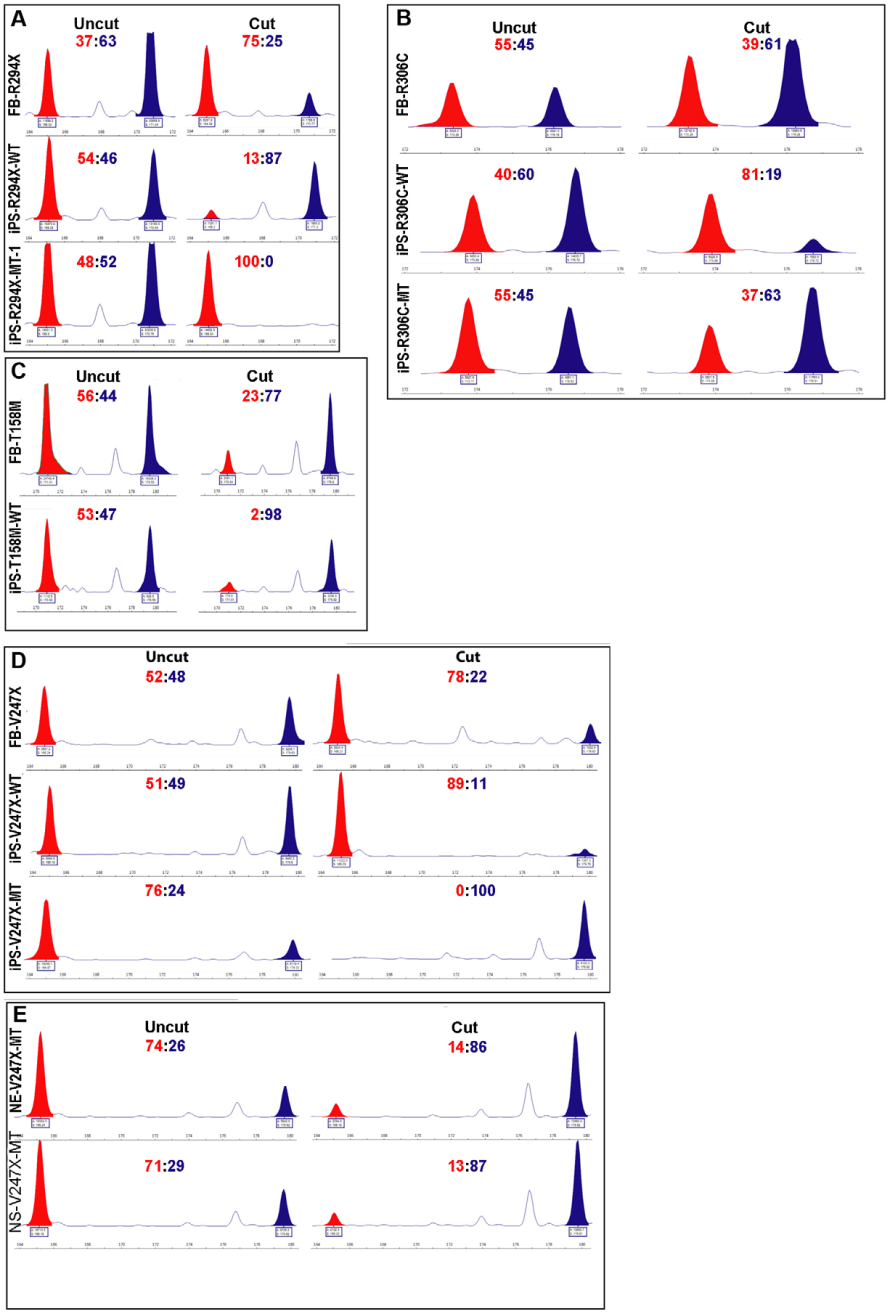
Nonrandom XCI pattern, as indicated by allele-specific methylation of the AR locus, in female human iPSC lines. The *AR* assay was used to examine the XCI pattern in RTT fibroblast and iPSC lines. (A) Results from the R294X fibroblast and iPSC lines. (B) Results from the R306C fibroblast and iPSC lines. (C) Results from the T158M fibroblast and iPSC lines. (D) Results from the V247X fibroblast and iPSC lines. (E) Results from the neuroepithelia (NE-V247X-MT) and neurospheres (NS-V247X-MT) differentiated from iPS-V247X-MT. Uncut traces detected presence of both parental *AR* alleles: 165 bp and 171 bp for the R294X lines; 173 bp and 176 bp for the R306C lines; 171 bp and 180 bp for the T158M lines; and 165 bp and 180 bp for the V247X lines. Cut traces only detected the hypermethylated *AR* allele on the inactivated X chromosome, which was resistant to digestion by the methylation-sensitive restriction enzyme HhaI. The source of genomic DNA was labeled to the left of each trace. The ratio between the two *AR* alleles was at the top of each trace. The molecular weight standard was provided under each trace.

**Figure 3:**
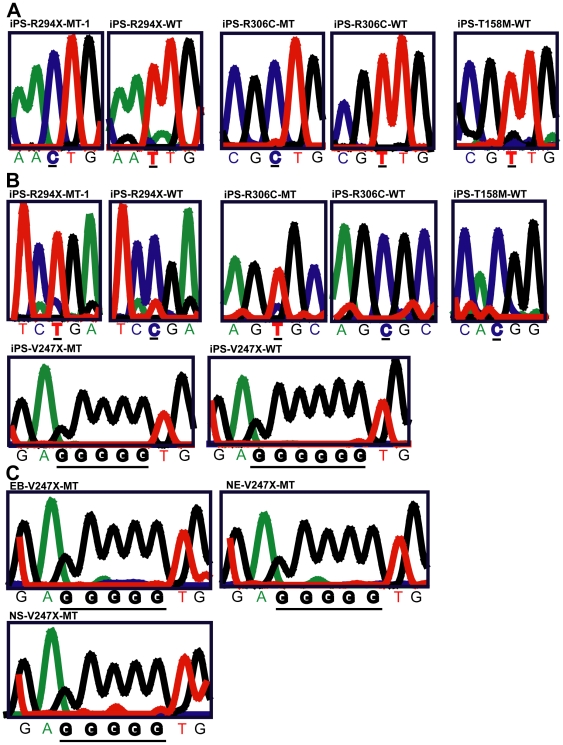
Clonal and allele-specific expression of *XIST* SNPs and *MECP2* in female human iPSC lines. (A) Representative sequencing traces of SNP analysis to distinguish allele-specific transcription of *XIST* in iPS-R294X-MT-1, iPS-R294X-WT, iPS-R306C-MT, iPS-R306C-WT, and iPS-T158M-WT. SNP rs1894271 (C or T) was examined in iPS-R294X-MT-1, iPS-R294X-WT. SNP rs16992442 (C or T) was examined in iPS-R306C-MT, iPS-R306C-WT, and iPS-T158M-WT. (B) Representative sequencing traces of regions of the *MECP2* gene that contain RTT mutations to distinguish allele-specific transcription of *MECP2* in iPS-R294X-MT-1, iPS-R294X-WT, iPS-R306C-MT, iPS-R306C-WT, and iPS-T158M-WT, iPS-V247X-MT, and iPS-V247X-WT. MT denotes expression of the mutant *MECP2* allele. WT denotes expression of the wild type *MECP2* allele. (C) Representative sequencing traces of the region of the *MECP2* gene that contains RTT mutation V247X (705delG) to distinguish allele-specific transcription of *MECP2* at the stages of embryoid body, neuroepithelia and neurosphere during directed neural differentiation from iPS-V247X-MT.

Female patients diagnosed with classic RTT are all heterozygous for the *MECP2* gene (disease causing gene) on the X chromosome. Due to random X chromosome inactivation (XCI), each somatic cell (including the fibroblasts) in these female RTT patients expresses either the wild type or the mutant, but not both, alleles of *MECP2* gene. Thus the XCI status in iPSCs derived from RTT patient fibroblasts directly determines which allele of *MECP2* is expressed in the neurons differentiated from these iPSCs. And the expression status of *MECP2* directly influences the interpretation of results from phenotypic analyses of neurons differentiated from RTT iPSCs. We performed several independent assays to determine the XCI status in all of our RTT iPSC lines. The first is a well-established methylation sensitive PCR assay to examine the methylation status of the androgen receptor (*AR*) locus on the X chromosome as a surrogate marker of XCI status [31], which has previously been used to examine XCI status in RTT patient brains [32] and RTT iPSCs [26], [27]. In this assay, without digestion with methylation sensitive enzymes (uncut traces), both parental alleles of *AR* can be readily detected and unequivocally distinguished by the presence of two different-sized peaks (red and blue) in all the original RTT fibroblast and iPSC lines (Figure 2). The non-skewed ratio between the red and blue peaks indicated similar amplification efficiency of the two parental alleles. After digestion with methylation sensitive enzymes (cut traces), only the hypermethylated *AR* allele (on the inactivated X chromosome) could be amplified. In all fibroblast lines, the ratio between the two parental *AR* alleles remained non-skewed, suggesting that XCI was random in the fibroblasts. In contrast, in all iPSC lines (except for iPS-R306C-MT), this ratio was highly skewed (13∶87 in iPS-R294X-WT, 100∶0 in iPS-R294X-MT-1, 81∶19 in iPS-R306C-WT, 2∶98 in iPS-T158M-WT, 89∶11 in iPS-V247X-WT, and 0∶100 in iPS-V247X-MT), suggesting XCI was not random in these female iPSCs. Furthermore, the same AR allele remained hypermethylated throughout directed neural differentiation from these iPSCs (Figure 2E and data not shown), indicating the XCI status remained unchanged from iPSCs to neurons. One of the R306C iPSC lines appeared to have less skewed XCI. This is most likely the result of insufficient separation of the smaller peak (173 bp) from the stutter peak (173 bp) of the bigger allele (176 bp). This technical hurdle is very difficult to overcome, because the two real peaks are only different by 3 base pairs. Similar difficulty in assaying the two *AR* alleles in the R306C iPSC lines has also been reported by the Ellis group [27]. In addition to the *AR* assay, we screened for heterozygosity of two single nucleotide polymorphisms (SNPs) in the *XIST* transcripts from the X chromosome. Both SNPs were recently described by the Plath group [30]. We found the R294X, R306C and T158M fibroblasts were heterozygous for one of the two SNPs in the *XIST* transcript. In the SNP assay, transcription of the two parental alleles of *XIST* in iPSCs generated from R294X, R306C and T158M fibroblast lines can be unequivocally distinguished by sequencing. These sequencing results again revealed nonrandom XCI in these female iPSC lines, because transcription from only one *XIST* allele could be detected in each line (Figure 3A). However, the allele-specific expression of *XIST* RNA does not necessarily mean that the inactive X chromosome is coated by *XIST* RNA in all cells. Finally, we directly examined allele-specific transcription of the *MECP2* gene in all the RTT iPSC lines described above. RT-PCR assays were performed to amplify fragments of the *MECP2* gene that contained the respective RTT mutations from RNA samples isolated from RTT iPSC lines. PCR amplicons were designed to span an intron to distinguish amplification of cDNA from that of genomic DNA. For missense and nonsense mutations, direct sequencing of the PCR fragments were performed to distinguish transcription from the mutant and the wild type alleles of the *MECP2* gene. For deletions (705delG in iPSC lines derived from GM07982, causing a frame shift and mutation V247X), individual clones of the PCR fragments were randomly chosen and sequenced to distinguish transcription from the mutant and the wild type alleles of the *MECP2* gene. In all RTT iPSC lines, we were able to detect transcription of either the wild type or the mutant, but not both, alleles of the *MECP2* gene (Figure 3B). This clonal pattern of *MECP2* transcription was maintained at several key stages (embryoid body, neuroepithelium, and neurosphere) throughout the process of directed neural differentiation from the iPSCs (Figure 3C). Collectively, our detailed analyses of 3 gene loci physically spread across the X chromosome (Figure S1) suggest all RTT iPSC lines generated in this study have nonrandom XCI and exclusively express either the wild type or the mutant allele of *MECP2*. Our results are consistent with two recent reports that showed iPSC generated from female human fibroblasts [30], including RTT patient fibroblasts [27], were clonal in XCI status. Taking advantage of the nonrandom XCI, we were able to screen and identify isogenic pairs of iPSC lines (expressing either the wild type or the mutant allele of *MECP2*) generated from the same RTT patient carrying the R294X, R306C, or V247X mutations. Similar to previously reported results [27], all iPSC lines derived from the T158M fibroblast expressed the wild type allele of *MECP2* (one example shown in Figure 3). The complete results of the XCI screening and the allele-specific transcription of *MECP2* were summarized in table S1.

### Neuronal differentiation and phenotypic characterization of one pair of isogenic RTT iPSC lines

**Figure 4:**
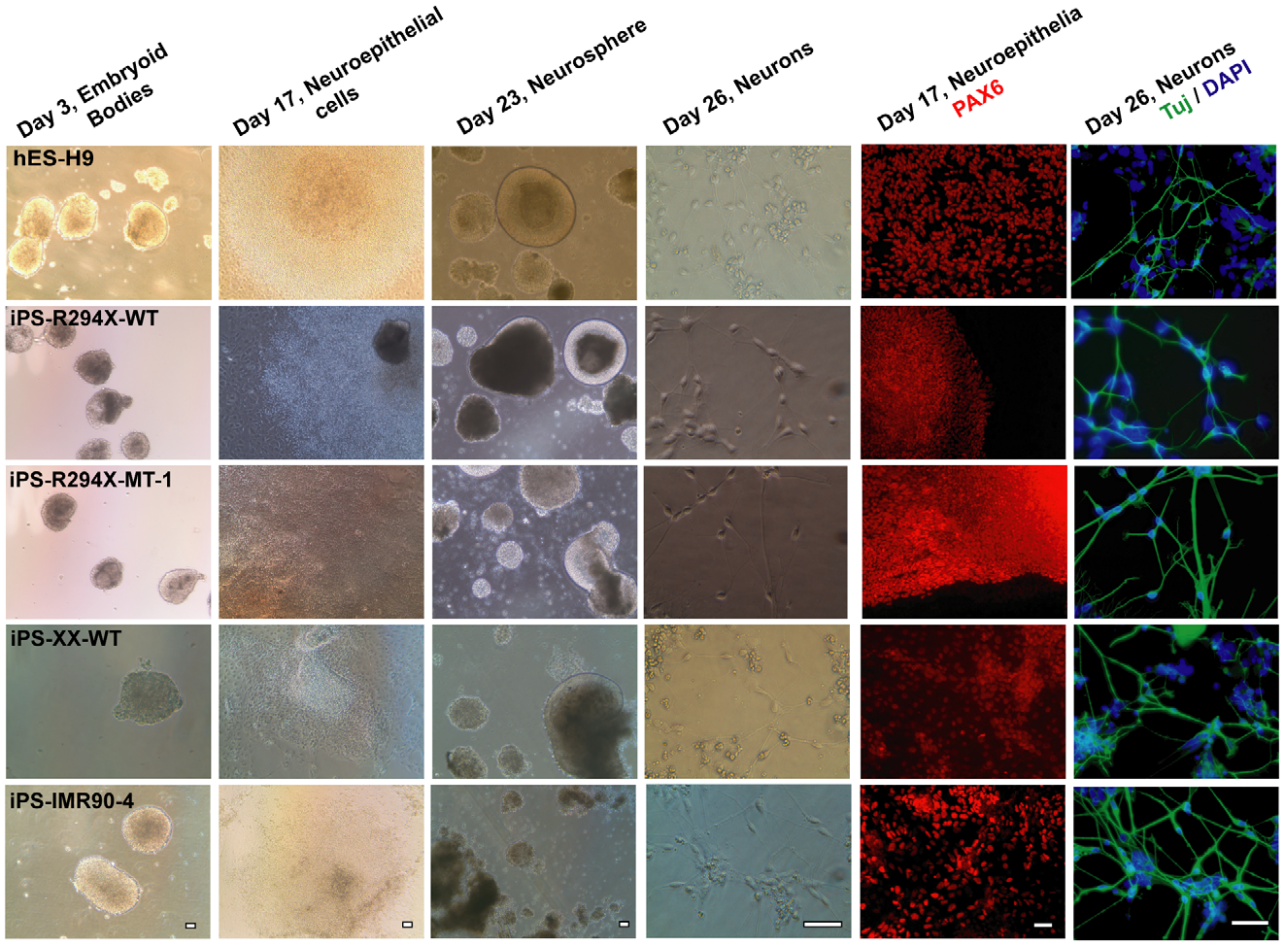
Isogenic pairs of wild type and mutant RTT iPSC lines efficiently differentiate into post mitotic neurons. Left 4 columns: representative transmitted light microscropy images at key stages of the directed neuronal differentiation. Each row represents differentiation from one hESC or hiPSC line. Each column represents one key stage of the differentiation. Right 2 columns: representative fluorescence images of expression of neuroepithelial marker PAX6 and postmitotic neuron marker beta III tubulin (Tuj). Each row represents differentiation from one hESC or hiPSC line. Scale bars = 200 µm.

Of the isogenic pairs of RTT iPSC lines generated in our study, R306C iPSCs have been phenotyped previously [26]. Although V247X iPSCs have not been studied before, this mutation is extremely rare. In addition, the clinical profile of the V247X RTT patient is not well established. Thus, we decided to focus our phenotypic analysis on the R294X isogenic pair, because this is a common RTT mutation with an established clinical profile. Moreover, RTT iPSCs with the R294X mutation have not been generated and characterized before. For directed neural differentiation, we adopted a well established and widely used protocol [33]. Briefly, embryoid bodies (EB) were formed *via* enzymatic disassociation of mature iPSC and hESC colonies from the mouse feeder layer and subsequent suspension culture. Seven days after dissociation, the EBs were plated down for differentiation into neuroepithelia (NE). At day 17, the neuroepithelial cells were mechanically dissociated and grown into neurospheres (NS) in suspension. At day 23, neurospheres were broken into small clusters and plated on laminin-coated surface to make neurons. Neurons migrating from the dissociated neurosphere clusters were visible ∼2 hours post plating. As expected, both iPS-R294X-WT and iPS-R294X-MT-1 line were able to differentiate into neurons at an efficiency comparable with those observed with H9 and iPS-IMR90-4 (Figure 4). Uniformity and high yields were achieved at key stages of the directed differentiation, including EB, NE, and NS (Figure 4 and data not shown). At the NE stage, characteristic neuronal rosettes exhibited uniform expression of Pax6 (Figure 4), a marker of neural progenitor cells. Neurons derived from the above describe iPSC lines and the hESC line H9 also showed robust expression of beta III tubulin, a marker for postmitotic neurons (Figure 4). No difference in differentiation efficiency was observed between iPS-R294X-WT and iPS-R294X-MT-1, which is consistent with the normal early/embryonic neuronal development observed in human RTT patients and in RTT mouse models.

**Figure 5:**
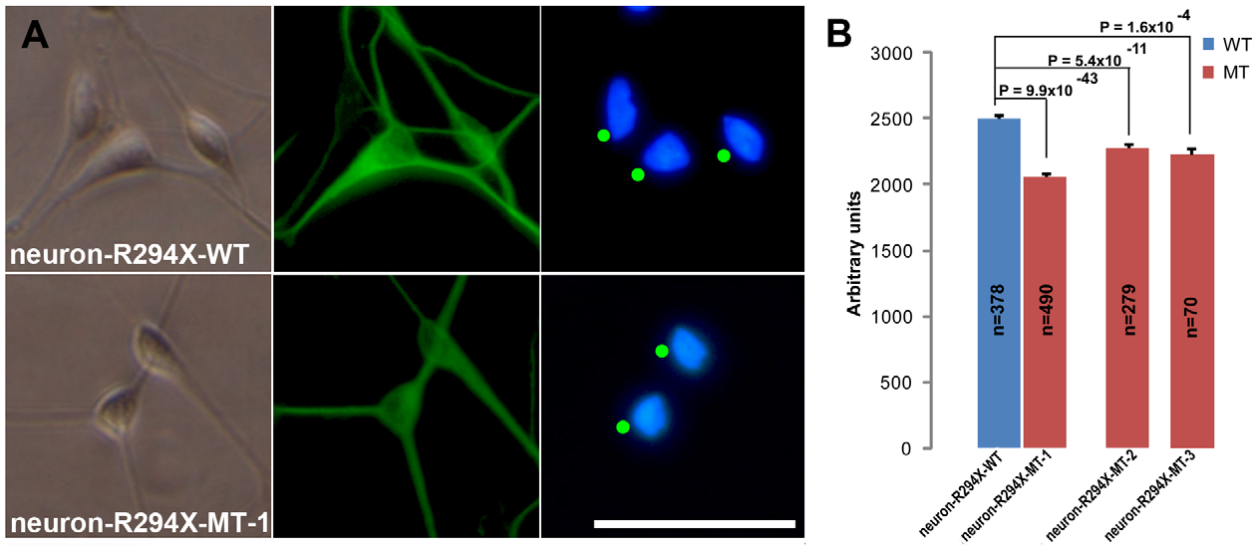
Analysis of nuclear size in neurons differentiated from isogenic pairs of R294X iPSC lines. (A) Representative high magnification images of neurons (neuron-R294X-WT and neuron-R294X-MT-1) derived from the isogenic pair of iPS-R294X-WT and iPS-R294X-MT-1 to demonstrate the characteristic neuronal morphology under transmitted light (left), which was used in combination of beta III tubulin immunoreactivity (middle) to identify neurons for measuring DAPI stained nuclear area (right). The green dots in the DAPI images indicate neuronal nuclei identified for size analysis. Scale bar = 200 µm. Scale bar applies to all panels in this figure. (B) Bar graph of neuronal nuclei area measurements using artificial units. Average nuclear area measurements from R294X neurons (red, neuron-R294X-MT-1, -2, and –3 were differentiated from three isogenic iPSC-R294X-MT lines, respectively, and analyzed 3 days after plating) and their isogenic controls (blue, neuron-R294X-WT was differentiated from the isogenic iPSC-R294X-WT line). Error bars represent standard error of the mean (SEM). The total number of nuclei measured in each line was shown on the bar. All p values are from two-tailed Student's t-tests.

One characteristic RTT pathology at the cellular level is the smaller brain/neuron size observed in RTT patients (as reviewed in [3]) and *Mecp2* mutant mice [9]. We next performed a detailed analysis of the nuclear size of neurons differentiated from iPS-R294X-WT and iPS-R294X-MT-1 (Figure 5A). We decided to quantify the nuclear size because it is easier to measure, its measurement is more reliable, and that it is well correlated with cell body size. As expected, the nuclear size of R294X neurons (neuron-R294X-MT-1) was 17% smaller than the isogenic wild type control neurons (Figure 5B, 2061±18 vs. 2493±24, p = 9.9×10^−43^). To rule out possible phenotypic variation caused by epigenetic differences among different iPSC lines, we differentiated two additional mutant iPSC lines from the same patient into neurons (neuron-R294X-MT-2 and neuron-R294X-MT-3), examined their nuclear size, and found that they are both ∼9% smaller than the isogenic wild type control neurons (Figure 5B, 2273±23 vs. 2493±24, p = 5.4.9×10^−11^ and 2267±50 vs. 2493±24, p = 1.6×10^−4^).

## Discussion

Two recent studies have reported the generation and phenotypic analyses of iPSCs from RTT patients. The Muotri group [26] generated iPSC lines from RTT patients and an unrelated healthy individual (control), observed random XCI in most of their RTT iPSC lines, and compared phenotypes in neurons differentiated from RTT iPSC lines and control iPSC and hESC lines. The Ellis group [27] generated iPSC lines from female RTT patients carrying mutations different from those included in the Muotri study, observed nonrandom XCI in all of their iPSC lines, and compared cell size in mutant neurons differentiated from an iPSC line with a rare deletion of almost the entire *MECP2* gene and their isogenic controls. These studies appear to contrast each other in the XCI status of female RTT iPSC lines. This is a critical issue because the *MECP2* gene, mutations in which cause RTT, is located on the X chromosome. Thus the XCI status in RTT iPSC lines directly determines which allele of *MECP2* is expressed in the neurons differentiated from these iPSC lines. And the expression status of *MECP2* directly influences the interpretation of results from phenotypic analyses of neurons differentiated from such RTT iPSC lines. In our study, we found nonrandom XCI in all of our female RTT iPSC lines. Our results are consistent with the finding of the Ellis group, as well as the original discovery of nonrandom XCI in female human iPSC lines [30]. To rule out potential difference in reprogramming procedures, we used two methods (Yamanaka factors delivered by retroviruses and Thompson factors delivered by lentiviruses) to generate iPSCs from 3 female RTT patients with common mutations (R294X, R306C, and T158M) and 1 female RTT patients with a rare mutation (V247X), and observed nonrandom XCI in a total of 23 iPSC lines regardless of the reprogramming methods used or the inherent RTT mutations. However, at this early stage of iPSC technology development, there are likely many unknown technical variations across different labs that may have caused the different XCI status in female iPSCs generated in these studies.

There may be certain advantages in using isogenic pairs of wild type and mutant disease-specific iPSC lines and their derivatives to studying disease mechanism. For instance, comparison within the isogenic pair may reduce the phenotypic variation across different iPSC lines from individuals with diverse genetic background. However, the failure to reactivate the inactivated X chromosome clearly demonstrates the incomplete reprogramming of the somatic epigenome, which may interfere with subsequent phenotypic analyses. As a first step to assess phenotypic variation caused by epigenetic difference among iPSC lines, we analyzed neurons differentiated from 3 isogenic mutant R294X iPSC lines. Our results showed that although there was variation among neurons from the three mutant lines, they were all significantly smaller than neurons differentiated from the isogenic wild type R294X iPSC line. Future analysis of additional isogenic wild type iPSC lines from the same patient will define the phenotypic variation among wild type lines, and conclusively answer the question of whether epigenetic difference among iPSC lines may mask genotype-dependent phenotypes.

To date, more than 200 mutations have been identified in the *MECP2* gene, all of which causes RTT. Since no clear genotype-phenotype correlation has been established between a certain mutation and the resulting spectrum of disease symptoms, it is necessary to generate and analyze RTT iPSC lines with different mutations (varying in the nature of mutation and the location of the mutation), when using RTT iPSCs and their derivatives to studying disease mechanisms. Compared with RTT iPSC that carry rare RTT mutation (such as the deletion mutation in [27]), our R294X isogenic pair carries a common RTT mutation that is identified in ∼5% of RTT patients. At the molecular level, the MeCP2^R294X^ protein may retain some of the important functions of the wild type protein (i.e. binding to methylated DNA because it still has the MBD domain), while lack other functions. Therefore, difference in the spectrum of phenotypes and the underlying mechanisms may exist between the R294X neurons and the *MECP2* null neurons described in [27].

In summary, we have established two novel isogenic pairs of RTT iPSC lines with either a common (R294X) or a rare (V247X) RTT mutation, and characterized the R294X pair. Together with the RTT iPSC lines generated by the Muotri group [26] and the Ellis group [27], these unique isogenic pairs of iPSC lines generated in our study are valuable tools for the RTT research community.

## Materials and Methods

All work related to generating iPSC from fibroblast lines from the Coriell Institute for Medical Research (Camden, NJ) has been approved by the Stem Cell Research Oversight (SCRO) Committee at the University of Wisconsin-Madison (protocol # SC-2009-0006), and has been exempted from Institution Review Board (IRB) review (IRB protocol # M-2009-1424).

The work related to generating iPSC from patient RS0502 has been approved by the IRB at University of Wisconsin-Madison (protocol # H-2009-0110). Written informed consent was obtained from patient RS0502/her guardian. All procedures were in accordance with the Helsinki Declaration and established guidelines at University of Wisconsin-Madison.

### Cell culture and generation of iPSCs

RTT patient (GM11270, GM17880, and GM07982) and an age-matched control female (AG07306) fibroblast lines were obtained from the Coriell Institute for Medical Research (Camden, NJ), and cultured according to the repository protocols. RS0502 fibroblast line was derived from a patient biopsy performed at the Waisman center (Madison, WI). Retroviruses containing the transcription factors *OCT4*, *SOX2*, *KLF4* and *c-Myc* [19] were used to generate iPSCs from fibroblast line RS0502. Lentiviruses containing the transcription factors *OCT4*, *SOX2*, *NANOG*, and *LIN28* [20] were used to generate iPSCs from all other fibroblast lines. Briefly, lentiviruses were produced and titered according to previously published protocols [34]. 1–2×10^5^ fibroblasts were transduced with a mixture of *OCT4*, *SOX2*, *NANOG*, and *LIN28* lentiviruses, and were plated on mouse embryonic fibroblasts. Cells were fed daily with hES media supplemented with 100 ng/ml recombinant zebra fish bFGF, or recombinant human FGF2. At day 10 cells were switch to MEF conditioned media supplemented with FGF2. Distinct iPSC colonies appeared as early as 21 days. The average time of colony picking was 30 days post infection. Initial colonies were mechanically picked and split for the first 4–6 passages. Following the initial mechanical splitting, cells were switched to hES media with 4 ng/ml FGF and passaged using 1 unit/ml dispase (Invitrogen). Media compositions were as follows. hES media: DMEM/F12 with 20% KOSR, 1× NEAA, 1× pen/strep, and 1 mM L-glutamine (all from Invitrogen) and 0.1 mM b-mercaptoethanol. Neuronal induction media (NIM): DMEM/F12 with 1% N2, 1× NEAA, 1× pen/strep and 2 µg/ml heparin (Sigma H3149). Neurobasal media (NBM): Neurobasal media with 1% N2, 2% B27 and 1× pen strep (all from Invitrogen).

### Neuronal differentiation

iPSCs and hESCs were differentiated to neurons as previously described [33]. Briefly, embryoid bodies (EB) were formed *via* enzymatic disassociation of mature iPSC and hESC colonies from the mouse feeder layer. The colonies were grown in suspension for four days and were fed daily with hES media without FGF. EBs were subsequently cultured in suspension for an additional three days, feeding every other day with Neuronal induction media (NIM). Seven days after dissociation, the EBs were plated down into six well tissue culture plates using NIM supplemented with 10% FBS (HyClone). 12 to 16 hours after plating, the FBS supplemented media was replaced with NIM without serum. The attached EBs were fed with NIM every other day for 10 days and allowed to differentiate into neuroepithelial cells. At day 17, the neuroepithelial cells were mechanically dissociated, and grown into neurospheres in a suspension of NIM with 2% B27. At day 23 to make neurons, neurospheres were incubated with accutase (Innovative Cell Technology) for 3 minutes at 37°C and broken into small clusters by mechanical force. Neurosphere particles were plated on 48 well tissue culture plates precoated with 20 µg/ml mouse laminin for 2 hours. Neurons migrating from the dissociated neurosphere clusters were visible ∼2 hours post plating.

### Immunocytochemistry

All cells were fixed for 20 minutes with 4% paraformaldehyde, and permeabilized for 30 minutes with 1% triton X100 (Sigma). Primary antibodies were incubated overnight at 4°C. Primary antibody dilutions are: anti-Oct4 1∶200 (Santa Cruz sc-5279), anti-Sox2 1∶500 (R&D systems AF2018), anti-NANOG 1∶500 (R&D systems AF1997), anti-Ssea4 1∶20 (DSHB), anti-Tra-1-81 1∶500 (Millipore MAB4381), anti-beta III tubulin 1∶500 (Sigma T8660), anti-Pax6 1∶5000 (DSHB). DAPI was 1∶10000.

### Neuronal nuclei measurement

Fixed neuronal cultures (3 days after plating) were immunostained for beta III tubulin, counterstained with DAPI, and imaged at 40× magnification. Neurons were identified by beta III tubulin immunoreactivity and characteristic neuronal morphology under transmitted light. The nuclear area was measured by circling the DAPI stained nuclei in Adobe Photoshop CS4 Extended.

### Karyotyping

G-band chromosome analysis of the iPSC lines was conducted by the cytogenetics lab at the WiCell Research Institute (Madison, WI).

### Teratoma formation

Teratoma formation and histological analysis of teratoma were conducted by the WiCell Research Institute (Madison, WI). A certified pathologist was consulted for identification of the three germ layers.

### Allele-specific transcription of *MECP2* and *XIST*

RNA was prepared using the SV Total RNA Isolation kit (Promega). cDNA was made using the qScript cDNA SuperMix (Quanta Biosciences), and used as PCR template to examine allele-specific transcription of *MECP2* and *XIST*. To eliminate the possibility of genomic DNA being amplified, the *MECP2* PCR primers were designed to span an intron. Primers: Forward GCAAAGCAGAGACATCAGAA, reverse 1 (for T158M, V247X) CAGATCGGATAGAAGACTCC, reverse 2 (for R294X, R306C) GCCCAGGGCTCTTACAGGTC. *XIST* SNP assay was performed as previously described [30]. Purified PCR products were directly sequenced.

### Bisulfite PCR analysis of the OCT4 promoter

Bisulfite conversion of genomic DNA was performed with the Ez-DNA Methylation kit (Zymo). A CpG island region of the OCT4 promoter was amplified using previously published primers and conditions [20]. Fragments were cloned using the pGEM-T Easy vector (Promega) and subsequently sequenced.

### The human androgen receptor assay

The human androgen receptor assay was preformed as previously described [31]. Briefly, 500 ng of genomic DNA was digested to completion with the methylation sensitive restriction enzyme Hha I. Both the cut and uncut samples were used as template in PCR reactions, using primers flanking the polymorphic repeats upstream of the human androgen receptor locus. PCR amplification was performed as previously described [27] using a forward primer labeled with 6-FAM on the 5′end. The fluorescently labeled PCR amplicons were resolved on a genetic analyzer. The GeneScan data was analyzed and the peak area a measured using ABI Peak Scanner software.

### Confirmation of retroviral transgene silencing

Retroviral transgene silencing was assessed *via* qPCR using primers and methodology as previously described [35]. RNA isolated from retrovirus infected fibroblasts three days after transduction was used as a reference control. RNA was prepared using the SV Total RNA Isolation kit (Promega). cDNA was made using the qScript cDNA SuperMix (Quanta Biosciences). A StepOnePlus real-time PCR system (Applied Biosystems) was used for amplification and quantification.

### Statistical analysis

For all statistical analyses in this report, an F-test was first performed to determine whether the samples had equal or unequal variance. Then the Student's t-test (2-tailed) was performed assuming either equal or unequal variance as determined by the F-test. The threshold of statistical significance was set at p = 0.01.

## Supporting Information

Figure S1 **Schematic drawings of the MeCP2 protein and the X chromosome.** Top: Schematic representation of the location of the RTT mutations in relation to the known functional domains within the MeCP2 protein. Bottom: Schematic representation of the physical location of the *AR*, *XIST* and *MECP2* loci on the X chromosome.
(TIF)

Figure S2 **Representative sequencing traces from genomic DNAs isolated from several iPSC lines generated from RTT patients to confirm these lines carry the same mutations and are heterozygous for these mutations as found in the original fibroblast cell lines.**
(TIF)

Figure S3 **Karyotyping of selected RTT iPS cell lines was performed at early (passage 6) and late (passage 24) passages for iPS-V247X-MT, mid passage (passage 14) for iPS-T158M-WT, and late passage (passage 21) for iPS-XX-WT.** Normal karyotype was observed in all of the lines.
(TIF)

Figure S4 **Demethylation of the** ***OCT4*** **promoter, an epigenetic characteristic of hESCs, was observed in iPSC lines generated in the current study.** The methylation status of the 5 CpG sites within the *OCT4* promoter in T158M fibroblasts and selected iPSC lines from the present study is shown. Each row of circles represents sequencing result from one clone of the bisulfite PCR product, while each circle represents a CpG site in the promoter. Open circles indicate unmethylated CpG, filled circles indicate methylated CpG, and grey circles indicate ambiguous calls regarding methylation status at that CpG.
(TIF)

Figure S5 **Teratomas were formed by subcutaneously injecting SCID mice with selected iPSC lines.** Shown are representative histology images of these teratomas. Each row represents images from one iPSC line as labeled on the left. Each column represents images of one tissue/germ layer as labeled on the top. All teratomas derived for the present study contained tissues developed from all three germ layers: cartilage/mesoderm, neuronal tissue/ectoderm and gut epithelium/endoderm.
(TIF)

Figure S6 **Silencing of retroviral transcription was examined in all R294X iPSC lines using realtime PCR.** Retroviral expression of reprogramming factors (Exo Oct4, Exo Sox2, Exo Klf4, and Exo cMyc) in retrovirus infected fibroblasts (FB+virus) was used as control and set as 1. Retroviral expression of these factors in uninfected R294X fibroblast (FB-R294X), human embryonic cell line H9 (hES-H9), and four R294X iPSC lines were normalized against the control. Data are presented as means ± SEM.
(TIF)

Table S1 **Summary of studies of XCI status screening and allele-specific expression of** ***MECP2*** **in multiple RTT iPSC lines.** WT: wild type allele of *MECP2*. MT: mutant allele of *MECP2*. Cut off for skewed is 80∶20. N/A: not applicable.
(DOC)
