# Supplementary figures and images for: Isogenic Pairs of Wild Type and Mutant Induced Pluripotent Stem Cell (iPSC) Lines from Rett Syndrome Patients as In Vitro Disease Model
Source: PLoS One. 2011 Sep 26;6(9):e25255. doi: 10.1371/journal.pone.0025255 (PMC3180386; doi:10.1371/journal.pone.0025255)

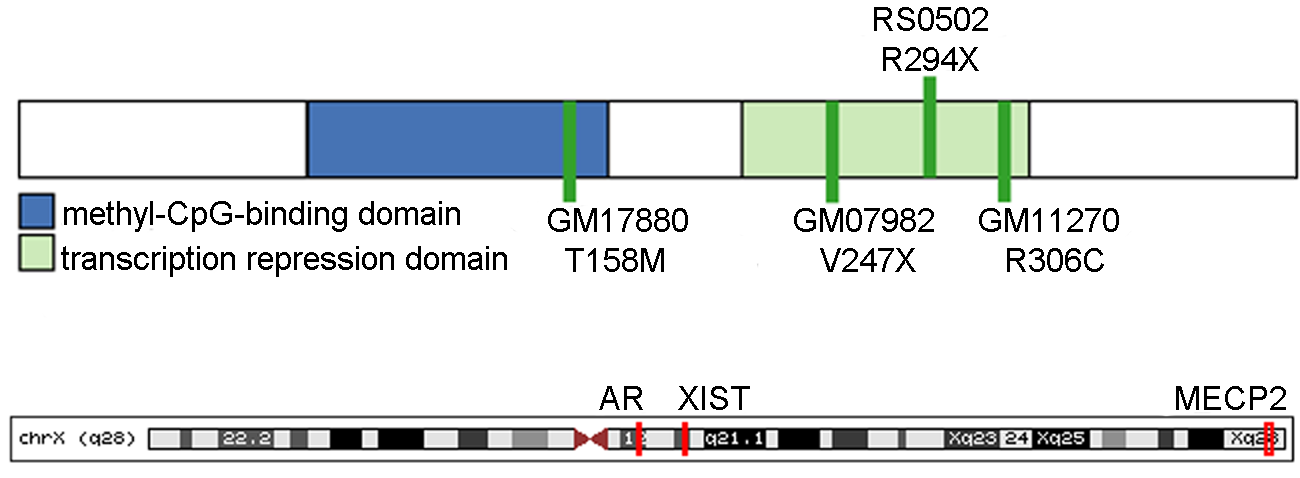

Supplement: Figure S1 — Schematic drawings of the MeCP2 protein and the X chromosome. Top: Schematic representation of the location of the RTT mutations in relation to the known functional domains within the MeCP2 protein. Bottom: Schematic representation of the physical location of the AR, XIST and MECP2 loci on the X chromosome. (TIF) [file pone.0025255.s001.tif]

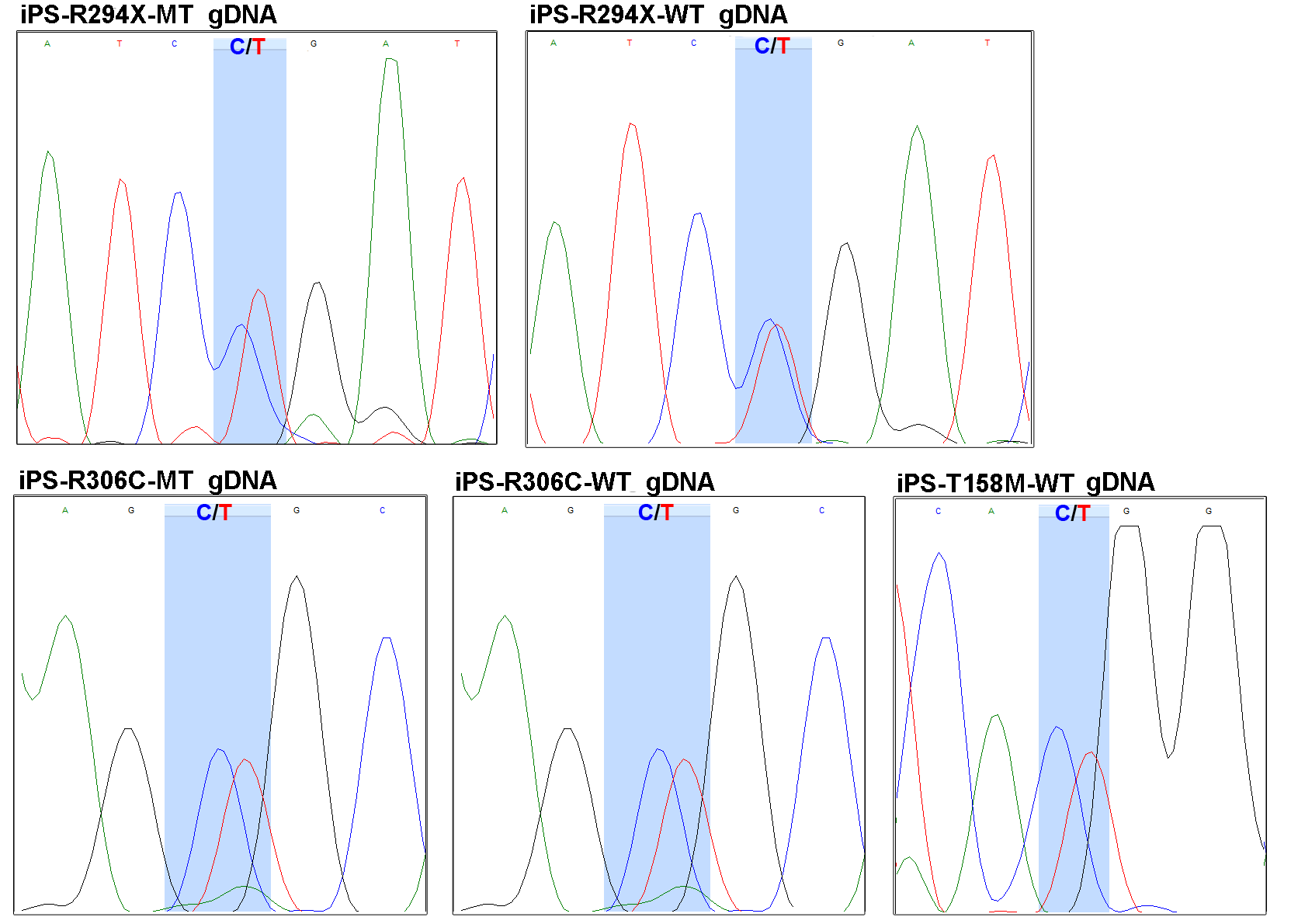

Supplement: Figure S2 — Representative sequencing traces from genomic DNAs isolated from several iPSC lines generated from RTT patients to confirm these lines carry the same mutations and are heterozygous for these mutations as found in the original fibroblast cell lines. (TIF) [file pone.0025255.s002.tif]

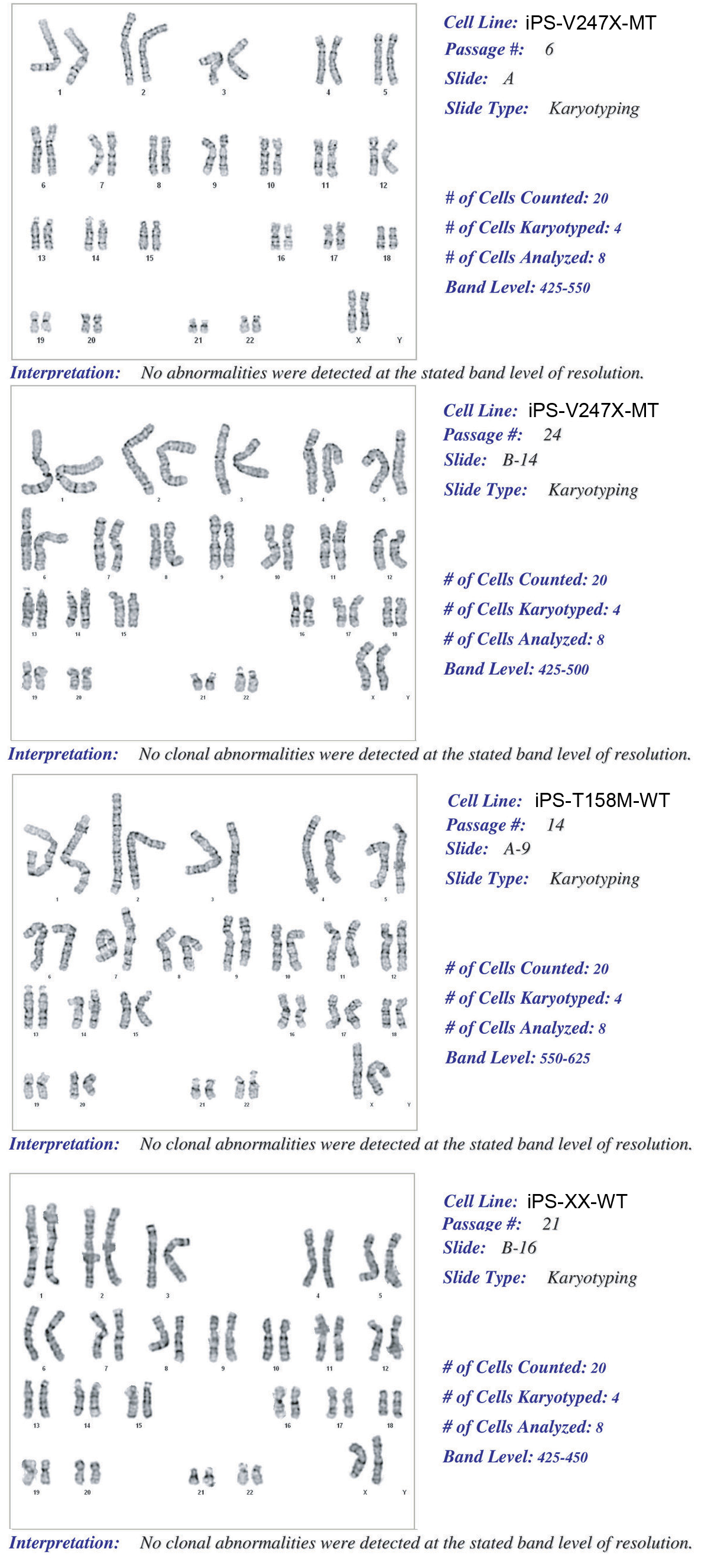

Supplement: Figure S3 — Karyotyping of selected RTT iPS cell lines was performed at early (passage 6) and late (passage 24) passages for iPS-V247X-MT, mid passage (passage 14) for iPS-T158M-WT, and late passage (passage 21) for iPS-XX-WT. Normal karyotype was observed in all of the lines. (TIF) [file pone.0025255.s003.tif]

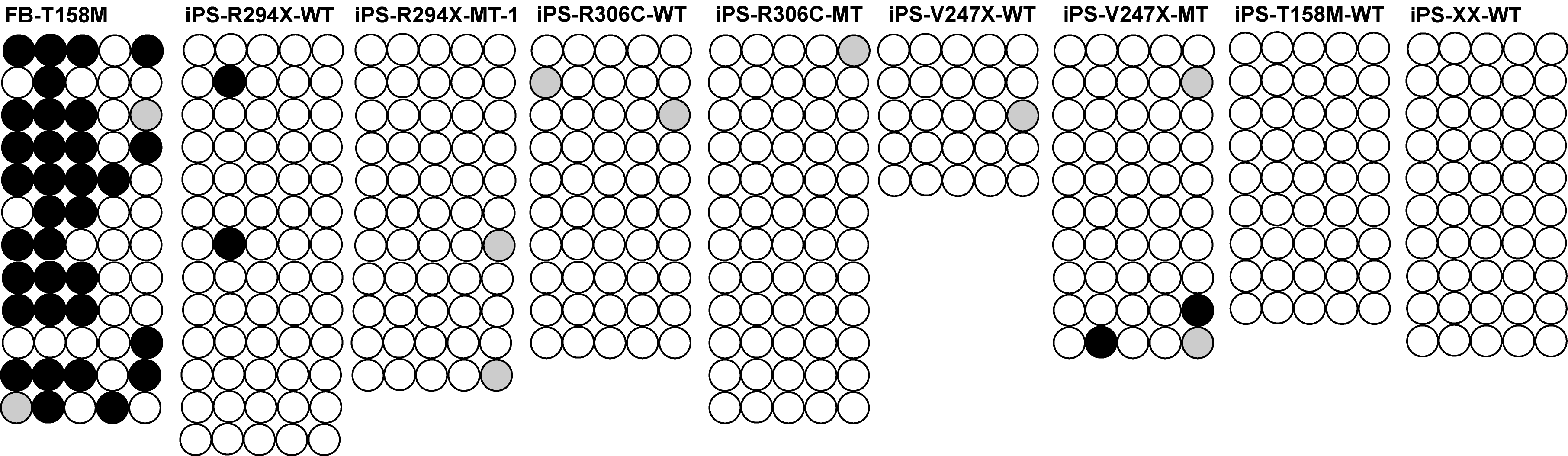

Supplement: Figure S4 — Demethylation of the OCT4 promoter, an epigenetic characteristic of hESCs, was observed in iPSC lines generated in the current study. The methylation status of the 5 CpG sites within the OCT4 promoter in T158M fibroblasts and selected iPSC lines from the present study is shown. Each row of circles represents sequencing result from one clone of the bisulfite PCR product, while each circle represents a CpG site in the promoter. Open circles indicate unmethylated CpG, filled circles indicate methylated CpG, and grey circles indicate ambiguous calls regarding methylation status at that CpG. (TIF) [file pone.0025255.s004.tif]

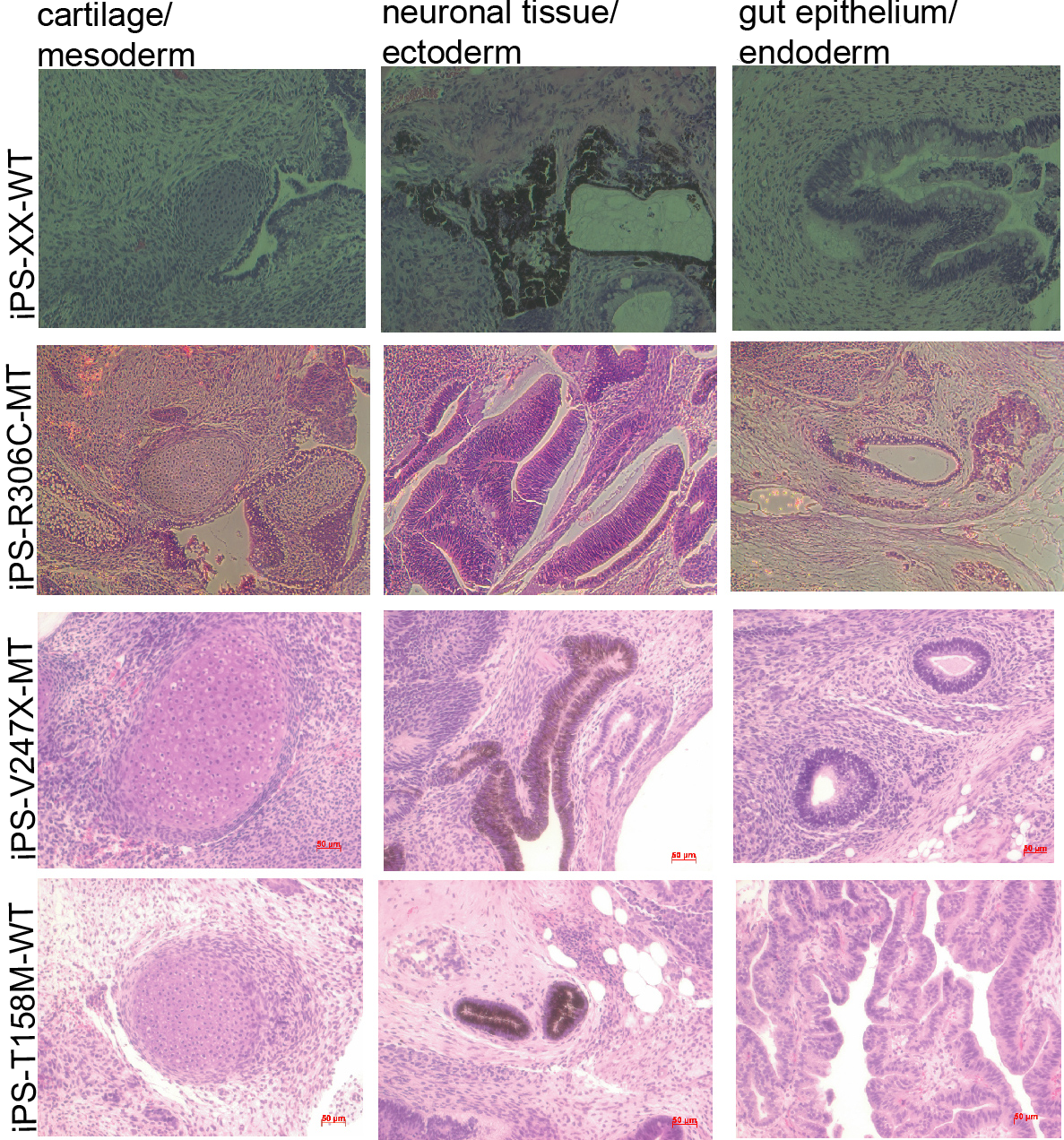

Supplement: Figure S5 — Teratomas were formed by subcutaneously injecting SCID mice with selected iPSC lines. Shown are representative histology images of these teratomas. Each row represents images from one iPSC line as labeled on the left. Each column represents images of one tissue/germ layer as labeled on the top. All teratomas derived for the present study contained tissues developed from all three germ layers: cartilage/mesoderm, neuronal tissue/ectoderm and gut epithelium/endoderm. (TIF) [file pone.0025255.s005.tif]

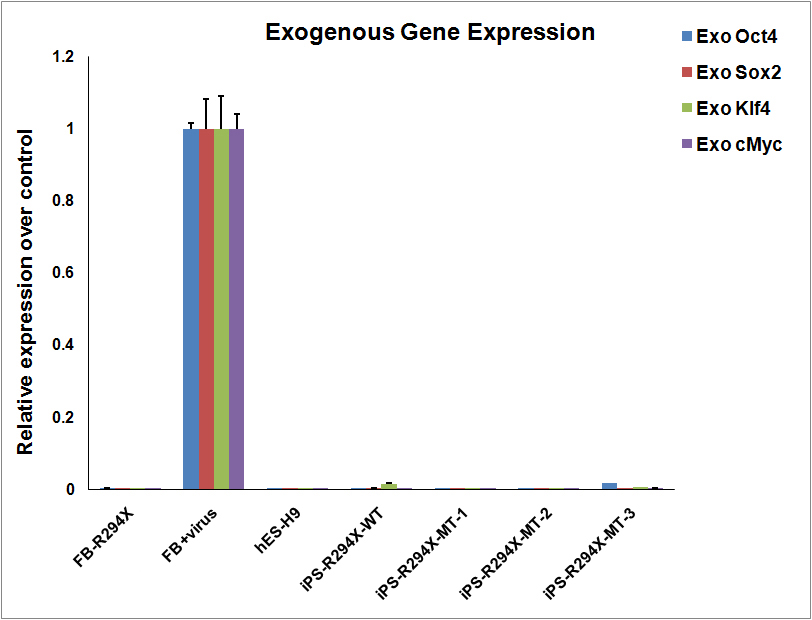

Supplement: Figure S6 — Silencing of retroviral transcription was examined in all R294X iPSC lines using realtime PCR. Retroviral expression of reprogramming factors (Exo Oct4, Exo Sox2, Exo Klf4, and Exo cMyc) in retrovirus infected fibroblasts (FB+virus) was used as control and set as 1. Retroviral expression of these factors in uninfected R294X fibroblast (FB-R294X), human embryonic cell line H9 (hES-H9), and four R294X iPSC lines were normalized against the control. Data are presented as means ± SEM. (TIF) [file pone.0025255.s006.tif]
