# Supplementary material for: Isogenic Pairs of Wild Type and Mutant Induced Pluripotent Stem Cell (iPSC) Lines from Rett Syndrome Patients as In Vitro Disease Model
Source: PLoS One. 2011 Sep 26;6(9):e25255. doi: 10.1371/journal.pone.0025255 (PMC3180386; doi:10.1371/journal.pone.0025255)
Supplement: Table S1 — Summary of studies of XCI status screening and allele-specific expression of MECP2 in multiple RTT iPSC lines. WT: wild type allele of MECP2. MT: mutant allele of MECP2. Cut off for skewed is 80∶20. N/A: not applicable. (DOC) [file pone.0025255.s007.doc]

**Supplementary Table 1: Summary of studies of XCI status screening and allele-specific expression of MECP2 in multiple RTT iPSC lines.**

| iPSC line | Allelic specific *MECP2* transcription | Allelic specific *AR* methylation | *XIST* SNP analysis | |
| --- | --- | --- | --- | --- |
|  |  |  | rs16992442 | rs1894271 |
| T158M-1 | WT | Not tested | T | N/A |
| T158M-3 | WT | skewed | T | N/A |
| T158M-4 | WT | Not tested | T | N/A |
| T158M-5 | WT | Not tested | Not tested | N/A |
| T158M-6 | WT | skewed | T | N/A |
| T158M-7 | WT | Not tested | T | N/A |
| T158M-8 | WT | Not tested | Not tested | N/A |
|  |  |  |  |  |
| V247X-1 | WT | skewed | N/A | N/A |
| V247X-2 | WT | skewed | N/A | N/A |
| V247X-4 | MT | skewed | N/A | N/A |
| V247X-5 | WT | skewed | N/A | N/A |
|  |  |  |  |  |
| R306C-1 | MT | Not tested | C | N/A |
| R306C-23 | MT | skewed | C | N/A |
| R306C-33 | WT | skewed | T | N/A |
| R306C-24 | WT | Not tested | Not tested | N/A |
| R306C-325 | MT | skewed | C | N/A |
|  |  |  |  |  |
| R294X-1 | MT | skewed | N/A | C |
| R294X-4 | MT | skewed | N/A | C |
| R294X-5 | MT | skewed | N/A | C |
| R294X-6 | MT | Not tested | N/A | C |
| R294X-10 | MT | Not tested | N/A | C |
| R294X-11 | WT | skewed | N/A | T |
| R294X-12 | MT | Not tested | N/A | C |

WT: wild type allele of *MECP2*. MT: mutant allele of *MECP2*. N/A: not applicable.
